# Decreased expression of the long non-coding RNA *FENDRR* is associated with poor prognosis in gastric cancer and *FENDRR* regulates gastric cancer cell metastasis by affecting fibronectin1 expression

**DOI:** 10.1186/s13045-014-0063-7

**Published:** 2014-08-29

**Authors:** Tong-peng Xu, Ming-de Huang, Rui Xia, Xin-xin Liu, Ming Sun, Li Yin, Wen-ming Chen, Liang Han, Er-bao Zhang, Rong Kong, Wei De, Yong-qian Shu

**Affiliations:** 1 Department of Oncology, the First Affiliated Hospital of Nanjing Medical University, No. 300 Guangzhou Road, Nanjing 210029, Jiangsu Province, People’s Republic of China; 2 Department of Medical Oncology, Huai’an First People’s Hospital, Nanjing Medical University, No. 6 Beijing Road West, Huai’an 223300, Jiangsu Province, People’s Republic of China; 3 Department of Biochemistry and Molecular Biology, Nanjing Medical University, Nanjing, Jiangsu Province, People’s Republic of China; 4 Department of Gastrointestinal Surgery, Subei People’s Hospital of Jiangsu Province, Yangzhou University, Yangzhou 225001, Jiangsu Province, People’s Republic of China

**Keywords:** Fibronectin1, FENDRR, Metastasis, Prognosis, Gastric cancer

## Abstract

**Background:**

*FENDRR* is a long non-coding RNAs (lncRNA) that binds to polycomb repressive complexe 2 (PRC2) to epigenetically regulate the expression of its target gene. The clinical role of *FENDRR* in carcinomas remains yet to be found.

**Method:**

Real-time polymerase chain reaction (PCR) was used to examine *FENDRR* expression in gastric cancer cell lines/tissues compared with normal epithelial cells/adjacent non-tumorous tissues. Cell proliferation assays, Wound healing assays, and in vitro and in vivo invasion and migration assays were performed to detect the biological effects of *FENDRR* in gastric cancer cells. Real-time PCR, western-blot and immunohistochemistry were used to evaluate the mRNA and protein expression of fibronectin1 (FN1). Secreted matrix metalloproteinase (MMP) activities were detected and characterized using gelatin zymography assay.

**Results:**

*FENDRR* was downregulated in gastric cancer cell lines and cancerous tissues, as compared with normal gastric epithelial cells and adjacent noncancerous tissue samples. Low *FENDRR* expression was correlated with deeper tumor invasion (p < 0.001), higher tumor stage (p = 0.001), and lymphatic metastasis (p = 0.007). Univariate and multivariate analyses indicated that low *FENDRR* expression predicted poor prognosis. Histone deacetylation was involved in the downregulation of *FENDRR* in gastric cancer cells. *FENDER* overexpression suppressed invasion and migration by gastric cancer cells in vitro, by downregulating FN1 and MMP2/MMP9 expression.

**Conclusion:**

Low expression of the lncRNA *FENDRR* occurs in gastric cancer and is associated with poor prognosis. Thus, *FENDRR* plays an important role in the progression and metastasis of gastric cancer.

## Background

Gastric cancer is the fourth most common malignancy in the world and is the second most frequent cause of cancer-related deaths worldwide, with particularly high incidence in East Asia [1],[2]. Although gastric cancer is curable if detected early, most patients are diagnosed in the advanced stage and have poor prognosis [3]. Tumor invasion and metastasis are critical steps in determining aggressive tumor phenotype and they also account for many cancer-related deaths [4]. The clinical stage, based on the TNM classification system, at the time of diagnosis is currently the most important prognostic factor, and the molecular mechanism involved in progression and metastasis of gastric cancer remains unclear [5]. Thus, novel findings on prognosis factors that are associated with gastric cancer progression and metastasis would be of great clinical relevance.

Apart from about 2% protein-coding genes, the vast majority of the human genome is made up of non-coding RNAs (ncRNAs),indicating that ncRNAs could play significant regulatory roles in complex organisms [6]. These non-coding regions are interspersed throughout genomic DNA. One subcategory of these transcripts, called long noncoding RNAs (lncRNAs), comprises ncRNAs that are more than 200 nucleotides in length. It is known that lncRNAs are widely transcribed in the genome, but our understanding of their functions is limited. Many studies have revealed that the deregulated expression of lncRNAs plays a functional role in a variety of disease states [7],[8]. In addition, recent reports have showed that some lncRNAs exhibit distinct gene-expression patterns and play significant roles during cellular development in various types of carcinomas [9]–[13]. However, the overall pathophysiological contributions of lncRNAs to gastric carcinoma remain largely obscure. Functional lncRNAs can be used for cancer diagnosis and prognosis, and serve as potential therapeutic targets; thus, lncRNAs can be considered as a new diagnostic and therapeutic gold mine in cancer [14].

The *FENDRR* gene is 3099nts in length, located at chr3q13.31, and consists of four exons. It is an lncRNA that is essential for proper heart and body wall development in mouse [15]. *FENDRR* can bind to both polycomb repressive complexe 2 (PRC2) and Trithorax group/MLL protein complexes (TrxG/MLL), which play pivotal roles in the control of chromatin structure and gene activity [16],[17]. *HOTAIR*, which is one of the few well-studied lncRNAs, plays a significant role in tumor progression by regulation of oncogene or tumor suppressor gene expression through binding to PRC2 [18]. Considering the role of HOTAIR, we hypothesized that *FENDRR*, another PRC2-binding lncRNA, may also be involved in tumorigenesis.

In this study, we found that *FENDRR* expression was reduced in gastric cancer tissues and cell lines. Low expression of *FENDRR* was associated with clinicopathological characteristics and poor prognosis in gastric cancer patients. Histone deacetylation contributed to the decreased expression of *FENDRR* in gastric cancer cells. Ectopic expression of *FENDRR* in gastric cells significantly inhibited cell migration and invasion. Conversely, depletion of *FENDRR* promoted these activities. Moreover, we found that fibronectin1 (FN1) and secreted matrix metalloproteinase (MMP) 2/ (MMP) 9 were involved in the *FENDRR*-mediated inhibition of cell migration and invasion. These results suggest *FENDRR* plays a significant role in the progression and metastasis of gastric cancer and could be used as a new therapeutic target.

## Results

### *FENDRR* expression was downregulated in gastric cancer tissues and cell lines, and histone deacetylation was involved in the downregulation of *FENDRR*

**Figure 1:**
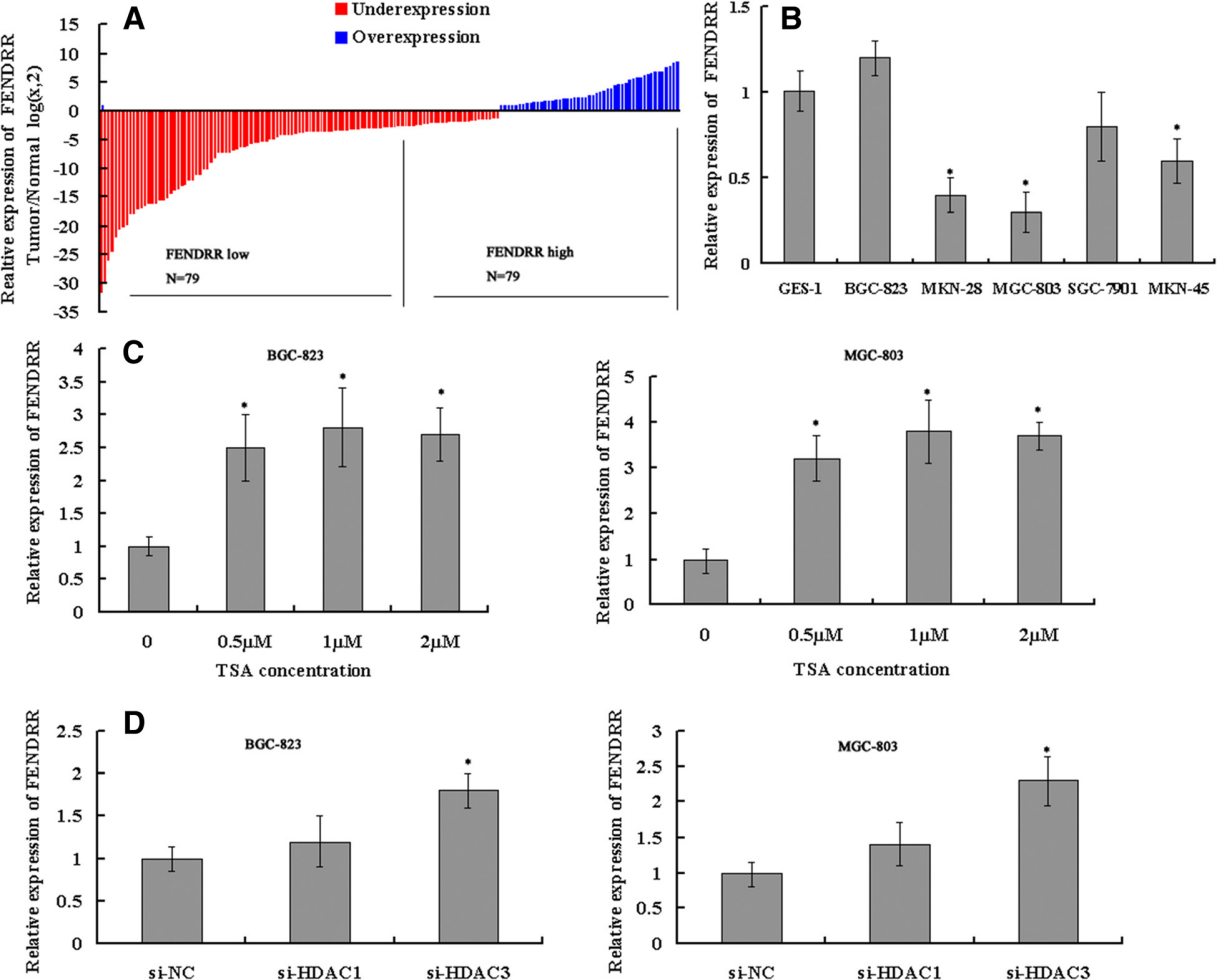
**Decreased expression of*FENDRR*in gastric cancer cells; histone deacetylation is involved in*FENDRR*downregulation. (A)***FENDRR* expression is examined by qRT-PCR in 158 paired human gastric cancer tissues and adjacent noncancerous tissues (Wilcoxon signed-rank test, p < 0.05). Data are represented as log_2_ fold change (cancer/normal), with “<−1” indicating underexpression, and “>1” indicating overexpression. The patients were divided into a low *FENDRR* expression group (79) and a high *FENDRR* expression group (79) according to the median value of relative *FENDRR* expression (2.7-fold, noncancerous/tumors) **(B)** Real-time PCR analysis of *FENDRR* expression in normal gastric epithelial cell line (GES-1) and gastric cancer cells. Experiments were performed in triplicate. Bars: SD; *p < 0.05. **(C)** qPCR analysis of *FENDRR* expression levels following the treatment of BGC823 and MGC803 cells with TSA. Experiments were performed in triplicate. Bars: SD; *p < 0.05. **(D)** qPCR analysis of *FENDRR* expression levels following the treatment of BGC-823 and MGC-803 cells with si-HDAC1 and si-HDAC3. Experiments were performed in triplicate. Bars: SD; *p < 0.05.

*FENDRR* expression levels were investigated in 158 paired gastric cancer samples and adjacent histologically normal tissues using quantitative polymerase chain reaction (qPCR) assays. *FENDRR* expression was significantly lower in tumor tissues than in the adjacent normal tissues (p < 0.05; Figure 1A). Reverse transcription (RT)-qPCR assays were further developed to quantify *FENDRR* in gastric cancer cell lines, including MGC803, BGC823, MKN28, MKN45 and SGC7901, and in the normal gastric epithelium cell line GES1. Significantly lower expression of *FENDRR* was found in MKN28 (p = 0.031), MKN45 (p = 0.041) and MGC803 (p = 0.035) than in GES-1, but there was no significant difference for BGC823 and SGC7901 (Figure 1B).

Next, we investigated the mechanisms controlling the tissue-specific expression of *FENDRR*. We analyzed the promoter and the first exon region of *FENDRR*, and found that there were two CpG islands. However, *FENDRR* expression was not changed after treatment with the DNA methyhransferase (DNMT) inhibitor 5-azacytidine (5-aza-C), indicating that DNA methylation contribution little to *FENDRR* expression (data not shown). Histone protein modification was thought to play an important role in the transcription of lncRNAs; however, the knockdown of two core subunits of PRC2 (SUZ12 and EZH2) had no influence on *FENDRR* expression (data not shown). Interestingly, *FENDRR* expression was induced in MGC803 and BGC823 cells after treatment with the histone deacetylase (HDAC) inhibitor trichostatin A (TSA) (Figure 1C). We sought to determine whether the inhibition of *FENDRR* was mediated by HDACs. Specific anti-HDAC1 and HDAC3 small interfering RNAs (siRNAs) were transfected into GC (gastric cancer) cells, and HDAC1 and HDAC3 expression was significantly decreased (Additional file 1: Figure S1). The expression levels of *FENDRR* were significantly upregulated in cells transfected with si-HDAC3 but not in those transfected with scrambled siRNA or si-HDAC1 (Figure 1D). These data indicate that the HDAC3 knockdown-mediated increase in *FENDRR* expression may be due to the inhibition of HDAC3 enzymatic activity.

### *FENDRR* expression and clinicopathologic factors in gastric cancer

To assess the correlation of *FENDRR* expression with clinicopathologic data, the expression levels of *FENDRR* in tumor tissues were categorized as low or high in relation to the median value of relative *FENDRR* expression (2.7-fold, noncancerous/tumors). Clinicopathologic factors were analyzed in the high and low *FENDRR* expression groups. As shown in Table 1, the low *FENDRR* group (n = 79) showed greater invasion depth (p < 0.001), higher tumor stage (p = 0.001), and more frequent lymphatic metastasis (p = 0.007) than the high *FENDRR* expression group (n = 79). However, there was no significant correlation between *FENDRR* expression and other clinicopathologic features such as age, sex, tumor location, tumor size, histologic grade, and distant metastasis (p > 0.05). Clinical data of all patients is shown in Additional file 2: Table S2.

**Table 1:** **Correlation between** ***FENDRR*** **expression and clinicopathological characteristics of gastric cancer**

**Clinical parameter**	***FENDRR***		**Chi-squared test P-value**
	**High No. cases (n = 79)**	**Low No. cases (n = 79)**	
Age (years)			0.426
<50	35	40	
>50	44	39	
Gender			0.255
Male	44	51	
Female	35	28	
Location			0.452
Distal	31	37	
Middle	28	28	
Proximal	20	14	
Size			0.152
>5 cm	37	46	
<5 cm	42	33	
Histologic differentiation			0.171
Well	4	6	
Moderately	34	21	
Poorly	34	41	
Undifferentiated	7	11	
Invasion depth			<0.001*
T1	30	6	
T2	28	13	
T3	11	34	
T4	10	26	
TNM Stages			0.001*
I	22	8	
II	32	23	
III	16	39	
IV	9	9	
Lymphatic metastasis			0.007*
Yes	33	50	
No	46	29	
Regional lymph nodes			<0.001*
PN0	47	28	
PN1	15	7	
PN2	10	27	
PN3	7	17	
Distant metastasis			0.797
Yes	8	9	
No	71	70	

*Overall p < 0.05.

### Low *FENDRR* expression is associated with poor prognosis of patients with gastric cancer

**Figure 2:**
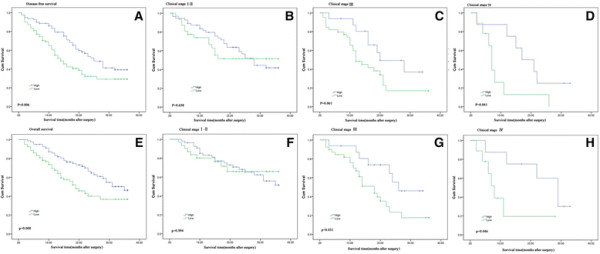
**The prognostic significance of*FENDRR*in gastric cancer patients. (A)** Kaplan–Meier analysis of disease-free survival (DFS) based on *FENDRR* expression in all 158 patients. **(B)**, **(C)**, and **(D)** Kaplan–Meier analysis of DFS based on *FENDRR* expression in gastric cancer patients in stages I-II **(B)**, III **(C)** and IV **(D). (E)** Kaplan–Meier analysis of overall survival (OS) based on *FENDRR* expression in all 158 patients. **(F)**, **(G)** and **(H)** Kaplan–Meier analysis of OS based on *FENDRR* expression in gastric cancer patients in stages I-II **(F)**, III **(G)** and IV **(H)**.

Kaplan–Meier analysis and log-rank test were used to evaluate the effects of *FENDRR* expression and the clinicopathological characteristics on disease-free survival (DFS) and overall survival (OS). The results showed that patients in the low *FENDRR* expression group had a higher recurrence rate (median DFS: 14 months) and much shorter overall survival (median OS: 19 months) than those in the high *FENDRR* expression group (median DFS: 25 months; median OS: 35 months; p = 0.006 and 0.008, respectively; Figure 2A,E).The 3-year DFS and OS were 29.2% and 36.7%,respectively, in the low *FENDRR* expression group, and 39.2% and 46.2%, respectively in the high *FENDRR* expression group. Moreover, the expression of *FENDRR* was strongly correlated with DFS and OS in the advanced clinical stages (stage III and IV; Figure 2D,G,H). However, in patients in clinical stages I and II, no significant difference in DFS and OS were found between those with low and high *FENDRR* expression (Figure 2B,F). Univariate analyses of clinical variables considered as potential predictors of survival are shown in Table 2. Further analysis in a multivariate Cox proportional hazards model showed that *FENDRR* expression, together with TNM stage, was strongly associated with OS. Furthermore, *FENDRR* expression was also significantly correlated with DFS in our study cohort. *FENDRR* expression was an independent prognostic indicator of DFS (hazard ratio [HR] = 0.555; 95% confidence interval [CI], 0.344–0.897; p = 0.016) and OS (HR = 0.569; 95% CI, 0.321–0.960; p = 0.042) in patients with gastric cancer (Table 2).

**Table 2:** **Univariate and multivariate Cox regression analyses** ***FENDRR*** **for DFS or OS of patients in study cohort (n = 158)**

**Variables**	**DFS**			**OS**		
	**HR**	**95% CI**	**p value**	**HR**	**95% CI**	**p value**
Univariate analysis						
Age (<50 years vs. >50 years)	0.935	0.618-1.415	0.752	0.954	0.599-1.520	0.843
Gender (male vs. female)	0.805	0.525-1.233	0.318	0.771	0.475-1.252	0.293
Location (Distal vs. Middle + Proximal)	0.827	0.546-1.254	0.372	0.714	0.448-1.138	0.157
Tumor size ( >5 cm vs. <5 cm )	1.392	0.917-2.113	0.120	1.236	0.975-1.565	0.079
Histologic differentiation(Well + Moderately vs. Poorly + Undifferentiated)	1.264	0.826-1.933	0.281	1.518	0.932-2.474	0.094
Invasion depth (T1 + T2 vs.T3 + T4)	1.306	0.863-1.978	0.207	1.323	0.829-2.111	0.240
TNM stage (I + II vs. III + IV)	0.470	0.308-0.717	<0.001*	0.405	0.251-0.653	<0.001*
Lymphatic metastasis (No vs. Yes)	0.617	0.404-0.924	0.025*	0.624	0.389-1.000	0.05*
Regional lymph nodes (PN2+ PN3 vs. PN0+ PN1)	0.626	0.410-0.954	0.029*	0.570	0.356-0.912	0.019*
Distant metastasis (No vs. Yes)	0.493	0.279-0.874	0.015*	0.523	0.275-0.996	0.049*
Expression of *FENDRR* (High vs. Low)	0.563	0.370-0.856	0.007*	0.539	0.337-0.862	0.010*
Multivariate analysis						
TNM stage (I + II vs. III + IV)	0.566	0.333-0.962	0.035*	0.458	0.253-0.828	0.010*
Lymphatic metastasis (No vs. Yes)	0.740	0.408-1.342	0.322	0.913	0.458-1.822	0.797
Regional lymph nodes (PN0+ PN1vs. PN2+ PN3)	0.658	0.338-1.283	0.220	0.779	0.363-1.674	0.522
Distant metastasis (No vs. Yes)	0.577	0.298-1.117	0.103	0.672	0.320-1.411	0.294
Expression of *FENDRR* (High vs. Low)	0.555	0.344-0.897	0.016*	0.569	0.321-0.960	0.042*

*Overall p < 0.05.

### *FENDRR* exhibits an insignificant effect on gastric cancer cell proliferation, but represses gastric cancer cell migration and invasion in vitro

**Figure 3:**
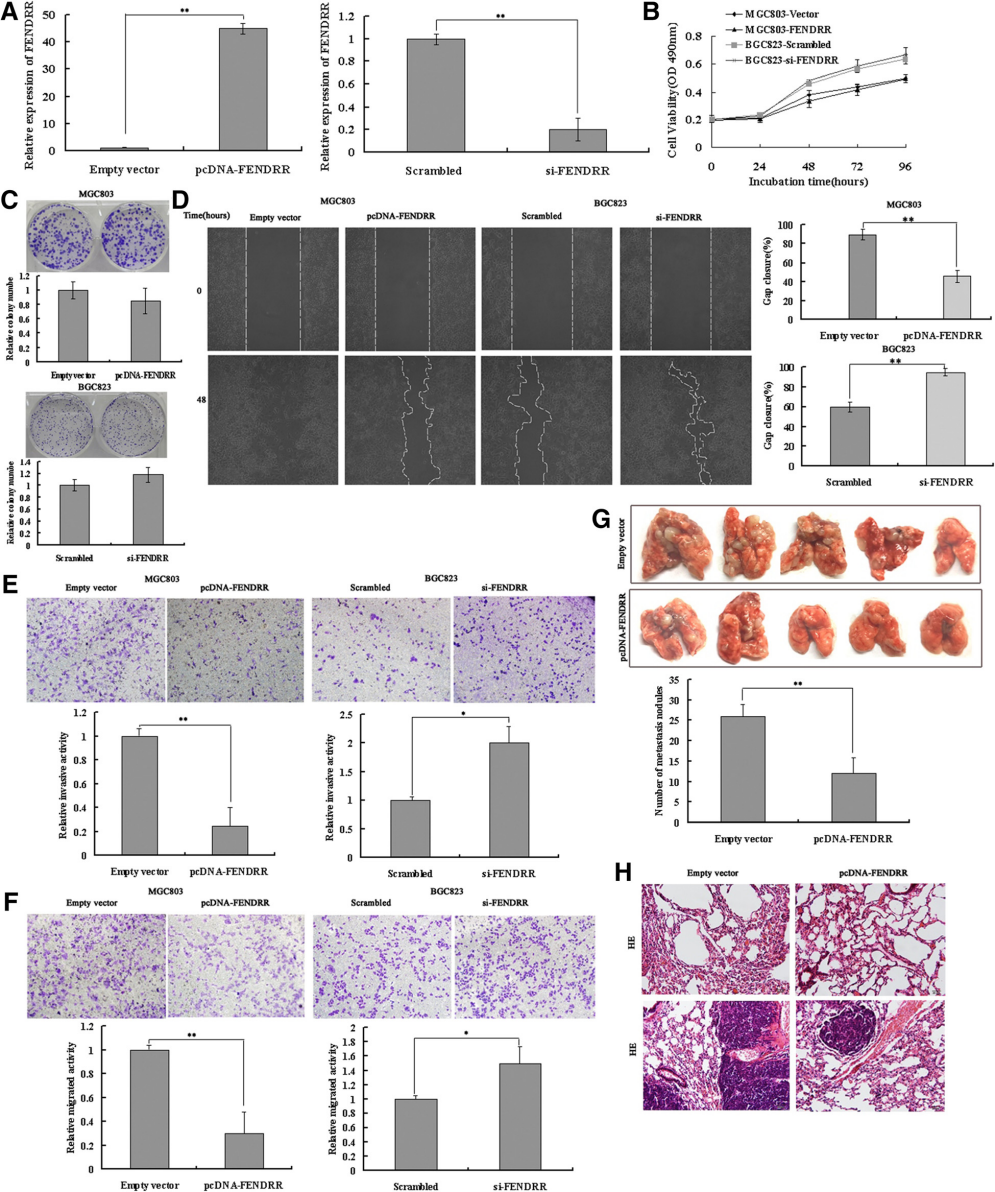
**Effects of*FENDRR*on gastric cancer cell migration and invasion in vitro and in vivo.** MGC803 cells were transfected with pcDNA-*FENDRR*, and BGC823 cells were transfected with si-*FENDRR*. **(A)** qPCR analysis of *FENDRR* expression levels following the treatment of MGC803 cells with empty vector and pcDNA-*FENDRR* (left panel), and the treatment of BGC823 cells with scrambled siRNA and si-*FENDRR* (right panel). Experiments were performed in triplicate. Bars: SD; **p < 0.01. **(B)** Forty-eight hours after transfection, MTT assays were conducted to determine the proliferation of MGC803 and BGC823 cells. Experiments were performed in triplicate. Bars: SD. **(C)** Colony-formation growth assays were conducted to determine the proliferation of MGC803 and BGC823 cells. The colonies were counted and photographed. Experiments were performed in triplicate. Bars: SD. **(D)** Wound healing assays were used to investigate the migratory ability of gastric cancer cells. Experiments were performed in triplicate. Bars: SD; **p < 0.01. **(E)** and **(F)** Transwell assays were used to investigate the changes in the migratory and invasive abilities of gastric cancer cells. Experiments were performed in triplicate. Bars: SD; *p < 0.05 and **p < 0.01. **(G)** MGC803 cells transfected with pcDNA-*FENDRR* and empty vector were separately injected into the tail veins of athymic mice. Lungs were harvested from the mice in each experimental group, and the numbers of tumor nodules visible on lung surfaces were counted. The assay was independently conducted twice. Bars: SD; *p < 0.05 and **p < 0.01. **(H)** Visualization of the entire lung, and hematoxylin and eosin (HE)-stained lung sections.

We further evaluated the role of *FENDRR* in cell proliferation, migration and invasion. *FENDRR* was over-expressed by transfecting pcDNA3.1-*FENDRR* vector into MGC803 cell line, which has a relatively low level of *FENDRR* expression. In addition, *FENDRR* was depleted in BGC823 cells, which exhibit a higher expression of *FENDRR.* The ectopic expression and knockdown of *FENDRR* in cells was confirmed by qRT-PCR. (Figure 3A).However, none of MTT assays and colony formation assays detected a significant proliferative effect of *FENDRR* in either the MGC803 or the BGC823 cell line (Figure 3B,C). Subsequently, we observed the effect on cell migration and invasion. As shown in Figure 3D, MGC803 cells, which have a naturally low *FENDRR* expression, when transfected for over-expression of *FENDRR* exhibited a notably lower scratch closure rate (migration inhibition) than observed in controls infected with empty vector. Moreover, BGC823 cells, which have a naturally high *FENDRR* expression, after knockdown of *FENDRR*, displayed a higher scratch closure rate (migration promotion) than control cells. Furthermore, cell motility was also measured using migration and invasion assay. Compared with the control cells, *FENDRR*-overexpressing MGC803 cells showed markedly repressed migration and invasion ability (p < 0.05; Figure 3E,F). Likewise, knockdown of *FENDRR* significantly stimulated migration and invasion of BGC823 cells (p < 0.05, Figure 3E,F). These findings indicate that *FENDRR* may be closely associated with invasion and migration of gastric cancer cell lines.

### *FENDRR* suppresses GC cell metastasis in vivo

To validate the effects of *FENDRR* on the metastasis of GC cells in vivo, MGC803 cells stably transfected with pcDNA-*FENDRR* were injected into nude mice. Metastatic nodules on the surface of lungs were counted after 7 weeks. Ectopic overexpression of *FENDRR* reduced the number of metastatic nodules compared with those in the control group (Figure 3G). This difference was further confirmed following examination of the entire lungs, and through hematoxylin and eosin (HE) staining of lung sections (Figure 3H). Our in vivo data complemented the results of functional in vitro studies involving *FENDRR*.

### Downregulated expression of FN1 and MMPs is involved in the *FENDRR*-mediated inhibition of gastric cancer cell metastasis

**Figure 4:**
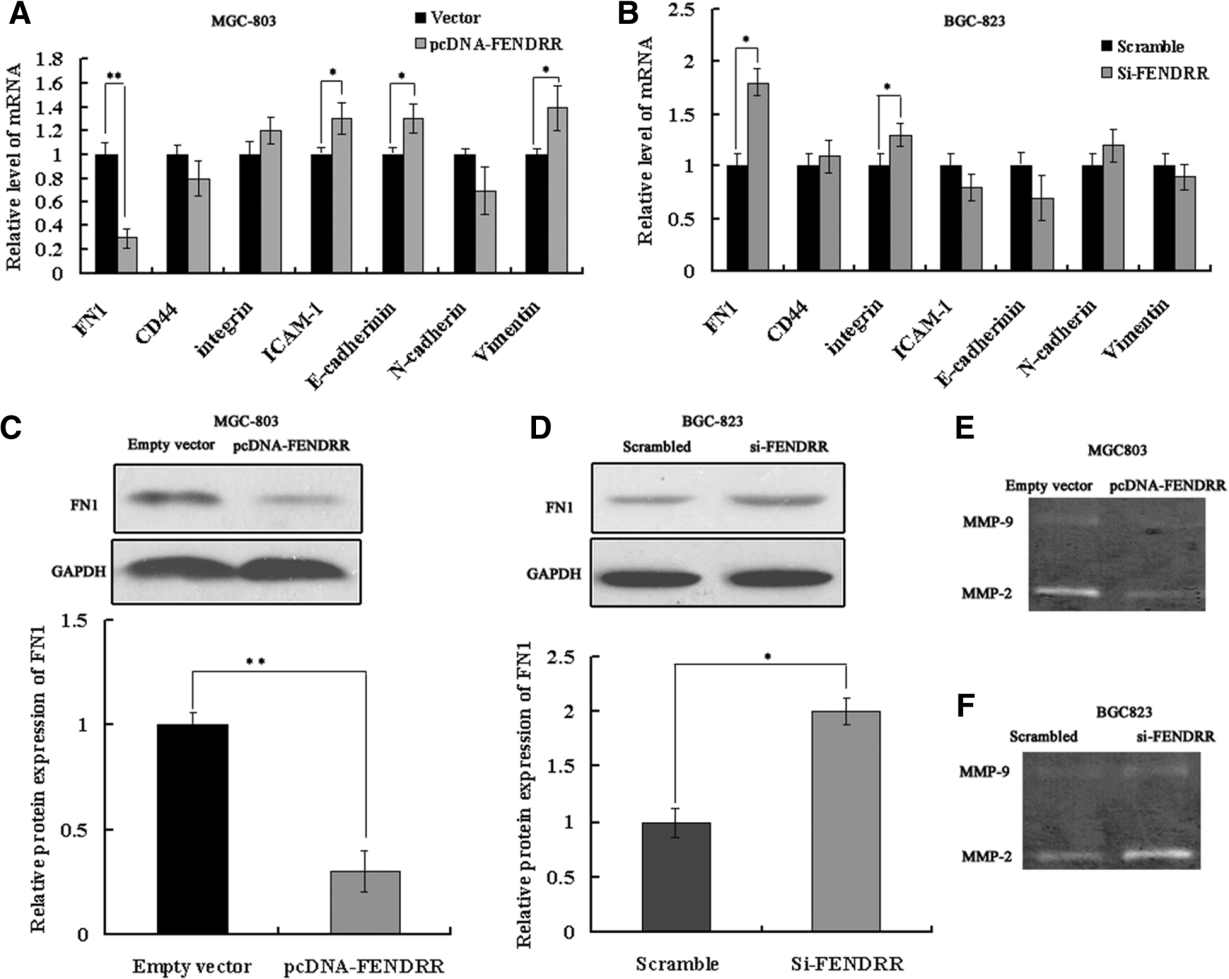
***FENDRR*regulates FN1 expression and MMP2/MMP9 activity. (A)** Expression of cell adhesion molecules (FN1, integrin, CD44, ICAM-1, E-cadherin, N-cadherin and vimentin) as detected using qRT-PCR after *FENDRR* was overexpressed in MGC803 cells or **(B)** blocked in BGC823 cells. **(C)** After *FENDRR* is overexpressed in MGC803 cells, western blot analysis shows that the FN1 protein level is diminished, as compared to the level in the control group. **(D)** The FN1 protein level is elevated after *FENDRR* expression is blocked in BGC823 cells, as compared to the level in the control group. **(E)** and **(F)***FENDRR* regulates the enzymatic activity of MMP2/MMP9 in gastric cancer cells. All the above experiments were performed in triplicate. Bars: SD; *p < 0.05 and **p < 0.01.

To explore the molecular mechanisms by which *FENDRR* contributes to the phenotypes of gastric cancer cells, we investigated potential targets involved in tumor invasion and metastasis. First, by using qRT-PCR, we detected the host gene *FOXF1*, which plays an important role in cancer cell invasion and migration [19], in *FENDRR*-overexpressing cells, *FENDRR*-knockdown cells, and control cells. However, no changed expression was found (data not shown). The result demonstrated that *FENDRR* may not exert its functions by regulating its host gene. Cell adhesion molecules are implicated in invasion and metastasis in various cancers*.* Therefore, we performed qRT-PCR to detect the expression of cell adhesion molecules that have been confirmed to be involved in tumor invasion and metastasis (such as, fibronectin1 [FN1], integrin, CD44, ICAM-1, E-cadherin, N-cadherin and Vimentin). Interestingly, fibronectin1 (FN1) was found to be significantly altered among them. When *FENDRR* was overexpressed or blocked, FN1 mRNA was diminished by 70% or elevated 2.0-fold, respectively, compared to the control groups (Figure 4A,B). Reports have shown that FN1 is associated with tumor migration and invasion [20]. Therefore, we determined the expression of the FN1 protein by performing western blot analysis. The FN1 protein level was also reduced by approximately 60% in MGC803 cells transfected with pcDNA-*FENDRR* (Figure 4C) and was elevated 2.0-fold after transfection of the cells with si-*FENDRR* (Figure 4D) compared to the respective controls. These data show that FN1 is negatively regulated by *FENDRR* at the mRNA and protein levels. MMPs are well known to contribute to cell invasion and metastasis in human carcinomas. We also found that the induction of *FENDRR* potently reduced the activity of MMP2/MMP9 in GC cells, and inhibition of *FENDRR* contributed to the activation of MMP2/MMP9, corroborating our earlier finding that *FENDRR* protected against gastric cancer cell metastasis (Figure 4E,F).

### FN1 knockdown also suppresses cellular migration and invasion in vitro

**Figure 5:**
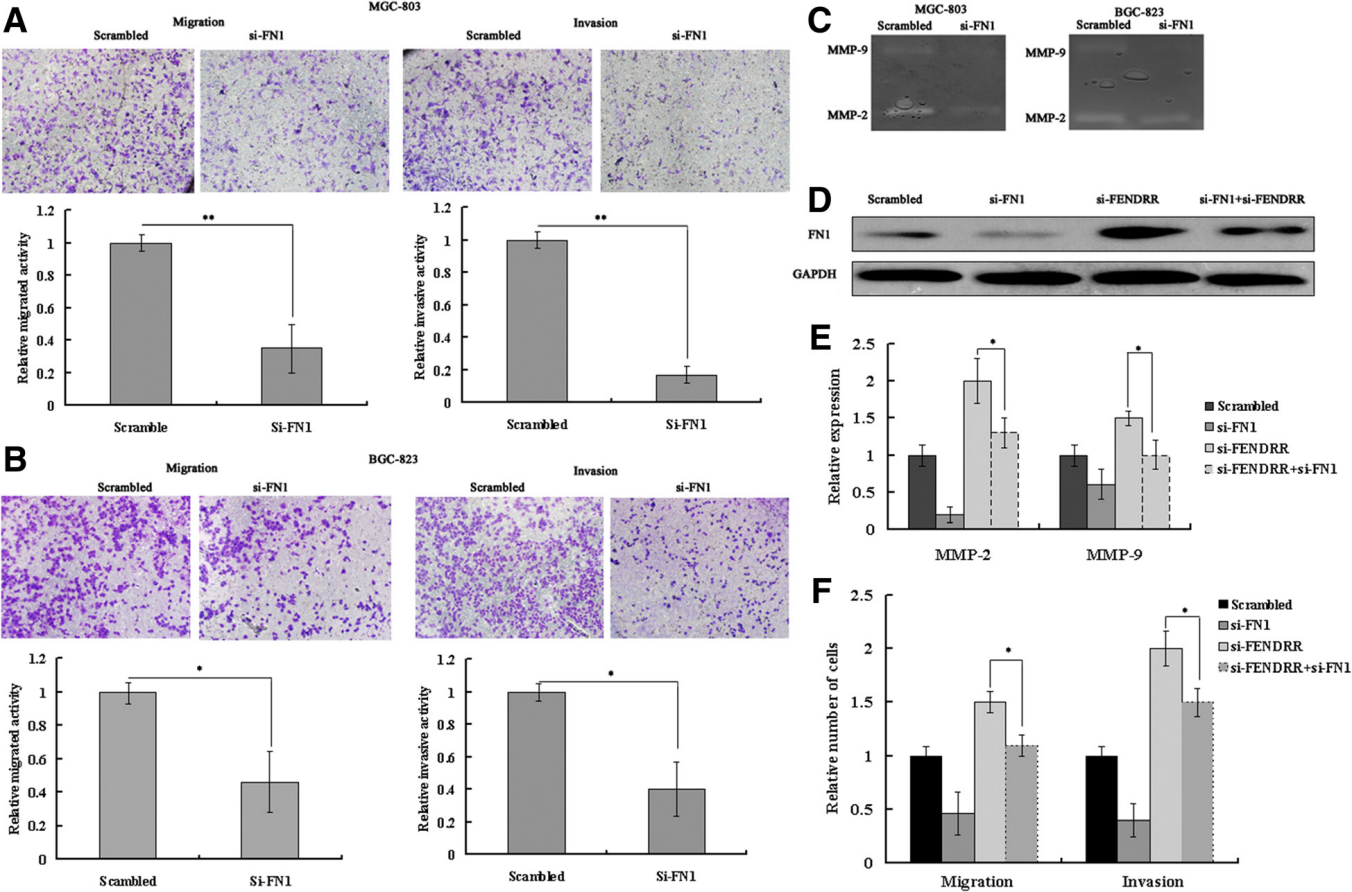
**FN1 promotes cells mobility and regulates MMP2/MMP9 activity in MGC803 and BGC823 cells; and is involved in the*FENDRR*-mediated inhibition of metastasis. (A)** and **(B)** FN1 knockdown inhibits the migration and invasion potential of MGC803 and BGC823 cells. **(C)** FN1 regulates the enzymatic activity of MMP2/MMP-9 in gastric cancer cells. **(D)** The expression of FN1 protein in BGC-823 cells was analyzed by Western blotting. **(E)** MMP2/MMP9 expression in BGC823 cells is analyzed by gelatin zymography. **(F)** Migration and invasion analyses of BGC823 cells is shown. All experiments were performed in triplicate. Bars: SD; *p < 0.05, **p < 0.01.

FN1 has been implicated in invasion and metastasis in diverse cancer; however, its role in gastric cancer is less well studied.. To determine the influence of FN1 on migration and invasion by gastric cancer cells, FN1 expression was knocked down by a siRNA targeting FN1 in MGC803 and BGC823 cells. Western blot and qRT-PCR analyses showed that transfection of gastric cancer cells effectively inhibited approximately 85% of FN1 expression at the mRNA level and 60% at the protein level, as compared to the expression in the control groups (Additional file 3: Figure S2A,S2B). Next, transwell assays examining cell migration and invasion were performed. On average, fewer si-FN1–transfected MGC803 and BGC823 cells than control cells were seen to migrate and invade (Figure 5A,B). A gelatin zymography assay revealed that depletion of FN1 also reduced MMP2/MMP9 expression (Figure 5C), which is consistent with previous reports [21],[22]. These results indicate that FN1 knockdown suppresses cell migration and invasion in gastric carcinoma cells, partially via downregulation of MMP2 and MMP9.

### Inhibition of FN1 is potentially involved in the tumor-suppressor function of *FENDRR*

To investigate whether FN1 was involved in the *FENDRR*–induced decrease in gastric cancer cell metastasis, we carried out rescue experiments. After transfection with si-FN1, BGC823 cells were cotransfected with si-FENDRR. We found that cotransfection of si-FN1 could rescue the upregulated expression of FN1 protein and MMP2/MMP9 induced by the depletion of *FENDRR* (Figure 5D,E). Moreover, migration and invasion assays indicated that the cotransfection could partially rescue si-*FENDRR*-promoted metastasis in BGC823 cells (Figure 5F). These data indicated that *FENDRR* inhibits gastric cancer cell migration and invasion partly through the downregulation of FN1 expression.

### Inverse relationship between the expression of FN1 and *FENDRR*

**Figure 6:**
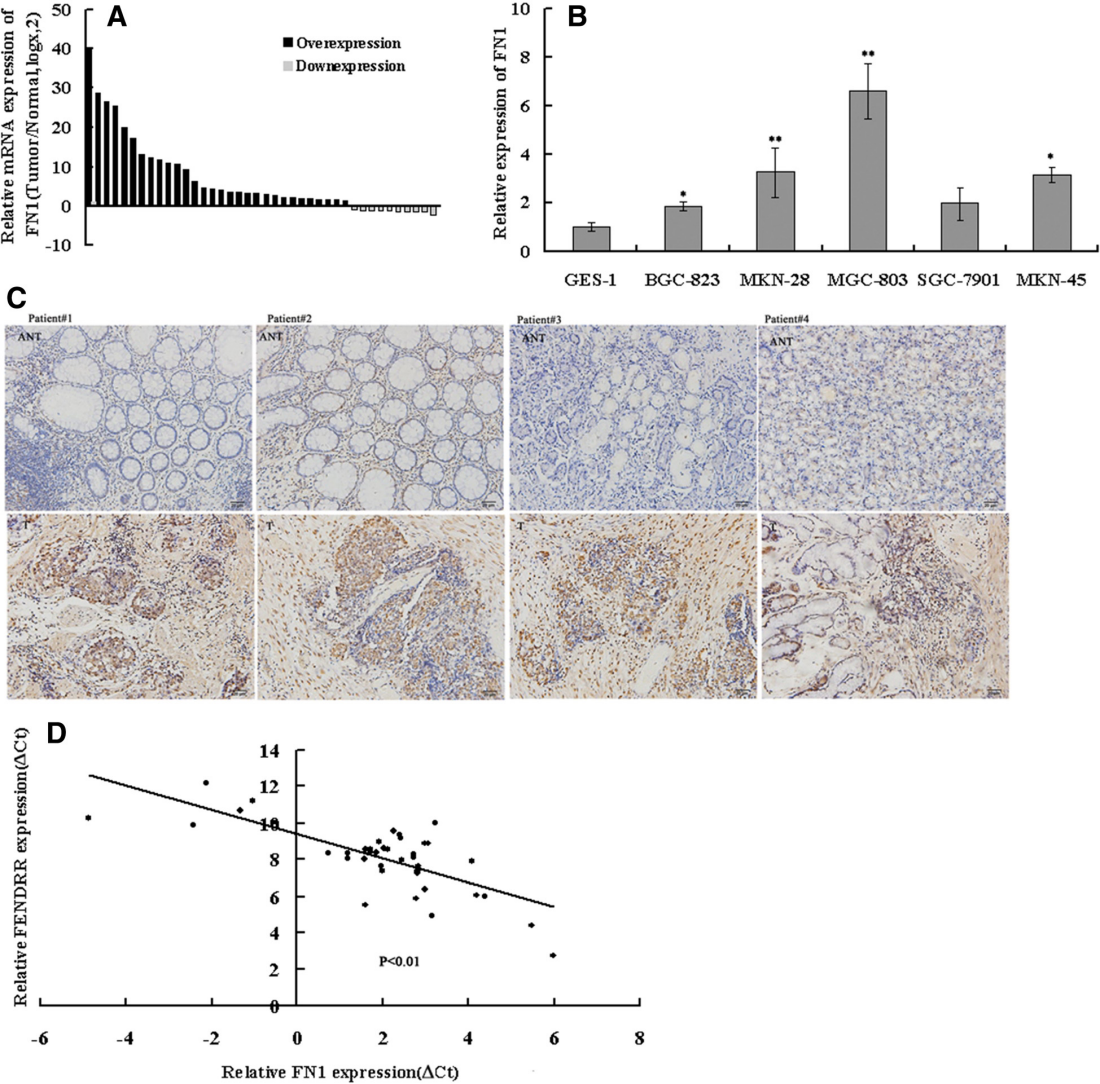
**FN1 expression in gastric cancer tissues and cell lines.** FN1 mRNA levels in gastric cancer tissues **(A)** and cell lines **(B)** were analyzed by qRT-PCR. **(C)** FN1 protein levels in gastric cancer tissues (T) and adjacent noncancerous tissues (ANT) were analyzed by immunohistochemistry. **(D)** Analysis of the relationship between FENDRR expression (△Ct value) and FN1 mRNA level (△Ct value) in 40 gastric cancer tissues. Bars: SD; *p < 0.05, **p < 0.01.

To assess the relationship between FN1 and *FENDRR* expression in gastric cancer, we examined FN1 expression by qPCR and immunohistochemistry in 40 paired gastric cancer tissues and in 5 gastric cancer cell lines. The results showed that the mRNA levels of FN1 were generally higher in gastric cancer tissues and cells, when compared with the levels in matched normal tissues and cells, respectively (Figure 6A,B).We also found that 80% of tumor-derived tissues showed higher FN1 protein levels, whereas most normal gastric tissues exhibited low FN1 protein levels, as compared with the levels in the paired tumor tissues (Figure 6C). Further analysis revealed that of *FENDRR* expression is inversely correlated with FN1 mRNA and protein levels in gastric cancer (Figure 6D, Additional file 4: Table S3). These data revealed that the FN1 level was mostly inversely correlated with the *FENDRR* level in gastric cancer.

## Discussion

Khalil et al. first identified the lncRNA *FENDRR* and confirmed its PRC2-binding functions [17]. Subsequently, Grote et al. found that it is an essential regulator of heart and body wall development [15]. However, its function in carcinogenesis and tumor progression is unclear. In this study, we first detected that *FENDRR* levels were decreased in gastric cancer cells and tissues compared with normal gastric epithelial cells and adjacent normal tissues. Moreover, low expression of *FENDRR* was significantly correlated with aggressive tumor characteristics (greater invasion depth, higher tumor stage, and lymphatic metastasis) and poor prognosis; when the patients were subdivided into three groups according to tumor stage, we found that *FENDRR* expression could better distinguish patients with different outcomes in stage III and IV. However, we did not observe a significant correlation between *FENDRR* expression and clinical outcome in early clinical stages of gastric cancer (clinical stages I–II, p = 0.638 for DFS and 0.994 for OS), probably due to good prognosis in the early stage of gastric cancer and the limited number of patients with stage I–II gastric cancer. Univariate and multivariate analysis indicated that OS and DFS were significantly better among patients with high *FENDRR* expression than in patients with low *FENDRR* expression in the same stage. Multivariate analysis demonstrated that *FENDRR* expression was an independent prognostic factor for gastric cancer patients. This suggests that *FENDRR* might be a promising prognostic biomarker in gastric cancer patients.

As low *FENDRR* expression was associated with an aggressive tumor phenotype in gastric cancer, we speculated that *FENDRR* could play a significant role in tumor biology. Initially, we chose representative cell lines of gastric cancer and investigated their *FENDRR* expression in comparison to a non-tumoral gastric cell line. We observed that 3 out of 5 tumoral cell lines exhibited low *FENDRR* expression, which corroborate our previous findings. We next determined whether *FENDRR* expression influenced tumor-like characteristics, such as migration, invasion, and metastasis. Indeed, ectopic expression of *FENDRR* inhibited cell migration and invasion, whereas knockdown of endogenous *FENDRR* expression significantly enhanced these capacities. Moreover, increased *FENDRR* expression significantly reduced the number of metastatic nodules on the lungs in vivo. However, no significant effect on cellular proliferation was observed after administration of ectopic expression or knockdown of *FENDRR*. This is in line with our clinical findings that *FENDRR* was significantly correlated with invasion depth, tumor stage, and lymphatic metastasis, but not tumor size. These results revealed that *FENDRR* might impact the prognosis of gastric cancer by affecting cell migration and invasion.

Many lncRNAs have been implicated in various types of cancers. Reportedly, the lncRNAs class *MALAT-1* has been found to promote cell motility in lung adenocarcinoma cells [23]. *PCGEM1* overexpression and *PRNCR1* have been identified for their involvement in prostate carcinogens [24],[25]. Gupta et al. [18] revealed that the lncRNA *HOTAIR* induces invasive and metastatic behavior in breast cancer cells. Tumor development and progression is precisely regulated by several subsets of genes that act by either silencing tumor suppressor genes or activating oncogenes [26]. In cancer cells, tumor-suppressor genes are usually silenced by genetic or epigenetic alterations [27]. Whether epigenetic regulatory factors, such as DNA methylation or histone acetylation, manipulate the expression of lncRNAs remains unkown. Hypermethylation of the promoter or the intergenic differentially methylated region has been found to contribute to reduced expression of lncRNA *MEG3* in tumors, indicating that epigenetic regulation is also involved in the expression of these genes [28]. Our findings emphasize that histone acetylation is a key factor in controlling the expression of the lncRNA *FENDRR*. These results, along with those from a recent study [28], highlight the role of epigenetics in regulating lncRNA transcription.

To explore the molecular mechanism through which *FENDRR* contributes to invasion and metastasis in gastric cancer, we investigated potential target proteins involved in cell motility and matrix invasion. We identified which genes were differentially expressed upon the ectopic expression or depletion of *FENDRR*, in comparison with untreated cells, FN1 mRNA levels were reduced or elevated after overexpression or blocking of *FENDRR*, respectively. Western blot analysis was performed to confirm that FN1 protein levels were also regulated by *FENDRR*. Importantly, the expression and activity of MMP2/MMP9 were also reduced upon *FENDRR* overexpression. FN1 is an extracellularmatrix glycoprotein that plays major roles in cell differentiation, growth and migration. It is involved in processes such as wound healing and embryonic development, as well as oncogenic transformation [29]. Importantly, FN is a key mediator of disease progression and metastasis in diverse carcinomas, such as skin squamous cell carcinoma [20], brain glioblastoma [30], and laryngeal squamous carcinoma [31]. For instance, FN and tissue transglutaminase 2 (TG2) contribute to the metastatic activity of A431 tumor cells, and this mediation may be partly due to the enhancement of FN and β integrin expression [20], FN1 is a key mediator of glioma progression through a mechanism that involves the maintenance of integrinβ1 FN receptors in glioma cells [32]. In this study, we found that FN1expression was upregulated in gastric cancer tissues and cell lines, when compared with the expression matched normal tissues and cells, respectively. We also confirmed that FN1 knockdown inhibits cell mobility in the gastric carcinoma cell line. Moreover, immunohistochemical analysis showed that the FN1 protein level was mostly inversely correlated with the *FENDRR* level in gastric cancer tissues, which indirectly confirmed that FN1 may be negatively regulated by *FENDRR*. MMPs are well known to play essential roles in invasion and metastasis in human carcinomas [33],[34]. FN1 has been reported to activate MMP2/MMP9 to promote invasion and metastasis in multiple carcinomas [21],[22],[35]. We showed that cotransfection with si-FN1 and si-*FENDRR* could partly “rescue” the MMP2/MMP9 upregulation induced by *FENDRR* downregulation, indicating that *FENDRR* regulated MMP2/MMP9 activity partly through FN1. However, the precise molecular mechanisms how *FENDRR* regulated FN1and MMPs remains unclear and is required further investigation.

The above evidence shows that histone deacetylation downregulates *FENDRR* expression in gastric cancer, and decreased *FENDRR* expression induces FN1 expression. Subsequently, the increased FN1 expression contributes to the activation of MMP2/MMP9, leading to higher migration and invasion potential of gastric cancer cells, which was manifested by the greater number of metastatic nodules in the nude mice. Moreover, our data also identified that patients exhibiting low *FENDRR* expression have higher metastasis potential and poor clinical outcomes.

## Conclusion

In summary, our study showed that *FENDRR* is dramatically downregulated in gastric cancer tissues and cell lines and that the low expression of *FENDRR* is significantly associated with invasion depth, tumor stage, lymphatic metastasis and patients’ survival time. Moreover, upregulation of *FENDRR* has the effect of suppressing gastric cancer cell migration and invasion in vitro by targeting FN1 and MMP2/MMP9. Further insights into the functional and clinical implications of *FENDRR* and its targets FN1 and MMP2/MMP9 may help with the treatment of gastric cancer.

## Materials and methods

### Cell lines

Human gastric adenocarcinoma cancer cell lines MGC803, BGC823, MKN28, MKN45 and SGC7901 and the normal gastric epithelium cell line (GES-1) were obtained from the Chinese Academy of Sciences Committee on Type Culture Collection cell bank (Shanghai, China). MGC803, BGC823 and MKN28 cells were cultured in RPMI 1640; MKN45, GES-1 and SGC7901 cells were cultured in DMEM (GIBCO-BRL) medium supplemented with 10% fetal bovine serum (FBS), 100 U/ml penicillin and 100 mg/ml streptomycin (Invitrogen, Carlsbad, CA, USA) at 37°C in 5% CO_2_.

### Tissue samples and clinical data collection

In this study, we analyzed 158 patients who underwent resection of the primary gastric cancer at the First Affiliated Hospital of Nanjing Medical University, Subei People’s Hospital of Jiangsu Province, and Huai’an First People’s Hospital of Jiangsu Province. The study was approved by the Ethics Committee on Human Research of the First Affiliated Hospital of Nanjing Medical University, Subei People’s Hospital of Jiangsu Province and Huai’an First People’s Hospital of Jiangsu Province and written informed consent was obtained from all patients. The clinicopathological characteristics of the gastric cancer patients are summarized in Table 1. All patients with gastric cancer have been followed up at intervals of 1–2 months until September 2013, and the median follow-up period was 36 months (range, 20–48 months). Follow-up studies included physical examination, laboratory analysis, and computed tomography if necessary. OS was defined as the interval between the dates of surgery and death. DFS was defined as the interval between the dates of surgery and recurrence; if recurrence was not diagnosed, patients were censored on the date of death or the last follow-up.

### RNA preparation and quantitative real-time PCR

Total RNAs were extracted from tumorous and adjacent normal tissues or cultured cells using Trizol reagent (Invitrogen) following the manufacturer’s protocol. RT and qPCR kits (Takara, Dalian, China) were used to evaluate the expression of *FENDRR* in tissue samples and cultured cells. The primers used in this study are shown in Additional file 5: Table S1. Real-time PCR was performed in triplicate, and the relative expression of *FENDRR* was calculated using the comparative cycle threshold (CT) (2^−ΔΔCT^) method with glyceraldehyde-3-phosphate dehydrogenase (GAPDH) as the endogenous control to normalize the data.

### Vector construction and transfection, and siRNA transfection

To overexpress *FENDRR*, the coding sequence of *FENDRR* was amplified and subcloned into the pcDNA3.1 (+) vector (Invitrogen) according to the manufacturer’ instructions. MGC803 cells were then transfected with a negative control vector or the *FENDRR*-expressing plasmid using Lipofectamine 2000 (Invitrogen). To generate *FENDRR*-knockdown BGC823 cells, the target sequence for *FENDRR* siRNA or scrambled siRNA that did not correspond to any human sequence was synthesized by Invitrogen. To generate FN1-knockdown BGC823 and MGC803 cells, the target sequence for FN1 siRNA was transfected into the cells, using Lipofectamine 2000 (invitrogen), according to the manufacturer’s instructions. The siRNAs si-HDAC1 and si-HDAC3 were transfected into BGC823 or MGC803 cells. The siRNA sequence were shown in Additional file 5: Table S1.

### Cell proliferation assays

Cell viability was monitored using a Cell Proliferation Reagent Kit I (MTT) (Roche Applied Science). The BGC823 cells transfected with si-*FENDRR* (3000 cells/well) and MGC803 cells transfected with pCDNA-*FENDRR* were grown in 96-well plates. Cell viability was assessed every 24 h following the manufacturer’s protocol. All experiments were performed in quadruplicate. For colony formation assays, pCDNA-*FENDRR*–transfected MGC803 cells (n = 500) were placed in a 6-well plates and maintained in media containing 10% FBS. The medium was replaced every 4 days; after 14 days, the cells were fixed with methanol and stained with 0.1% crystal violet (Sigma-Aldrich). Visible colonies were then counted. For each treatment group, wells were assessed in triplicate, and experiments were independently repeated three times.

### Wound healing assay

For the wound healing assay, 3 × 10^5^ cells were seeded in 6-well plates, cultured overnight, and transfected with pCDNA-*FENDRR*, si-*FENDRR* or a control. Once cultures reached 85% confluence, the cell layer was scratched with a sterile plastic tip and washed with culture medium. The cells were then cultured for 48 h with medium containing 1% FBS. At different time points, images of the plates were acquired using a microscope. The distance between the two edges of the scratch was measured using the Digimizer software system. The assay was independently repeated three times.

### Cell migration and invasion assays

For the migration assays, at 48 h post-transfection, 5 × 10^4^ cells in serum-free media were placed into the upper chamber of an insert (8-μm pore size; Millipore). For the invasion assays, 1 × 10^5^ cells in serum-free medium were placed into the upper chamber of an insert coated with Matrigel (Sigma-Aldrich). Medium containing 10% FBS was added to the lower chamber. After incubation for 24 h, the cells remaining on the upper membrane were removed with cotton wool. Cells that had migrated or invaded through the membrane were stained with methanol and 0.1% crystal violet, imaged, and counted using an IX71 inverted microscope (Olympus, Tokyo, Japan). Experiments were independently repeated three times.

### Western blot assay and antibodies

Cells were lysed using RIPA protein extraction reagent (Beyotime, Beijing, China) supplemented with a protease inhibitor cocktail (Roche, CA, USA) and phenylmethyl-sulfonyl fluoride (Roche). The concentration of proteins was determined using a Bio-Rad protein assay kit. Protein extracts (50 μg) were separated by 10% sodium dodecyl sulfate-polyacrylamide gel electrophoresis (SDS-PAGE), transferred to nitrocellulose membranes (Sigma) and incubated with specific antibodies. Electrochemiluminescence (ECL) chromogenic substrate was used to visualize the bands and the intensity of the bands was quantified by densitometry (Quantity One software; Bio-Rad), with GAPDH used as a control. Antibodies (1:1000 dilution) against FN1 were purchased from BD.

### Gelatin zymography

Conditioned media were obtained after a 30-h incubation of the different BGC823 and MGC803 cells in serum-free medium and were then concentrated 80-fold using Amicon Ultra Centrifugal Filter Units (Millipore) and normalized by protein concentration. Samples were loaded on 10% SDS-PAGE gels containing 0.1% gelatin. Electrophoresis was carried out under nonreducing conditions at 100 V and 4°C. The gels were then washed in 2.5% Triton X-100, incubated in substrate buffer (50 mmol/L Tris–HCl, pH 8.0; 50 mmol/L NaCl; 10 mmol/L CaCl_2_; and 0.05% Brij 35) for 40 h at 37°C, stained with Coomassie stain solution (Bio-Rad), and destained in 20% methanol and 10% acetic acid. Gelatinolytic activity was identified as a clear band on a blue background. The activities of secreted MMPs were detected using gelatin zymography as previously described [36], with several modifications.

### Tail vein injections into athymic mice

Male athymic mice (4-weeks-old) were purchased from the Animal Center of the Chinese Academy of Science (Shanghai, China) and maintained in laminar flow cabinets under specific pathogen-free conditions. BGC823 cells transfected with pCDNA-*FENDRR* or the empty vector were harvested from 6-well plates, washed with phosphate-buffered saline (PBS), and resuspended at a density of 2 × 10^7^ cells/ml. The cell suspension (0.1 ml) was injected into the tail veins of 10 mice, which were sacrificed 7 weeks after the injection. The lungs were removed and photographed, and visible tumors on the lung surface were counted. This study was carried out in strict accordance with the Guide for the Care and Use of Laboratory Animals of the National Institutes of Health. Our protocol was approved by the Committee on the Ethics of Animal Experiments of Nanjing Medical University (Permit Number: 200933). All surgery was performed under sodium pentobarbital anesthesia, and all efforts were made to minimize suffering [37]. The assays were independently performed for two replicates.

### Immunohistochemical (IHC) analysis

The immunohistochemical analysis of FN1 was performed according to a previously described method [38]. To quantify FN1 protein expression, both the intensity and extent of immunoreactivity were evaluated and scored. In the present study, staining intensity was scored as follows: 0, negative staining; 1, weak staining; 2, moderate staining; and 3, strong staining. The scores of the extent of immunoreactivity ranged from 0 to 3, and were determined according to the percentage of cells that showed positive staining in each microscopic field of view (0, <25%; 1, 25%–50%; 2, 50%–75%; 3, 75%–100%). A final score ranging from 0 to 9 was achieved by multiplying the scores for intensity and extent.

### Statistical analysis

All statistical analyses were performed using SPSS 20.0 software (IBM, SPSS, Chicago, IL, USA). The significance of the differences between groups was estimated by the Student t-test, χ2 test, or Wilcoxon test, as appropriate. DFS and OS rates were calculated by the Kaplan–Meier method with the log-rank test applied for comparison. Survival data were evaluated using univariate and multivariate Cox proportional hazards models. Variables with a value of p < 0.05 in univariate analysis were used in subsequent multivariate analysis on the basis of Cox regression analyses. Kendall’s Tau-b and Pearson correlation analyses were performed to investigate the correlation between *FENDRR* and FN1 protein expressions. Two-sided p-values were calculated, and a probability level of 0.05 was chosen for statistical significance.

## Abbreviations

lncRNA: Long non-coding RNA

HDAC: Histone deacetylase

TSA: Trichostatin A

GC: Gastric cancer

DFS: Disease-free survival

OS: Overall survival

HR: Hazard ratio

FN1: Fibronectin

MMPs: Matrix metalloproteases

## Competing interests

The authors declare that they have no competing interests.

## Authors’ contributions

XTP designed the study, detected the cells biological function, conducted the qRT-PCR assays, carried out the Western blotting assays, established the animal model, performed the statistical analysis, performed the immunohistochemistry assays, and drafted the manuscript. HMD and LXX provided the tissue samples and the clinical data, XR participated in the design of the study, and administrated the Gelatin zymography assays, SM, CWM, HL, YL, ZEB, KR, and DW helped to acquire experimental data. SYQ conceived the study, participated in its design and coordination, and helped to draft the manuscript. All authors read and approved the final manuscript.

## Additional files

## Supplementary Material

Additional file 1: Figure S1. qPCR analysis of HDAC1 and HDAC3 expression levels following the treatment of BGC823 and MGC803 cells with si-HDAC1 and si-HDAC3. Bars: SD; *p < 0.05, **p < 0.01. Click here for file

Additional file 2: Table S2. Clinical data of all patients involved in the study. Click here for file

Additional file 3: Figure S2. MGC803 and BGC823 cells were transfected with an siRNA targeting FN1, causing the suppression of the mRNA **(A)** and protein levels **(B)** of FN1. Bars: SD; *p < 0.05, **p < 0.01. Click here for file

Additional file 4: Table S3. Relative *FENDRR* expression level and FN1 scores on immunohistochemistry. Click here for file

Additional file 5: Table S1. Primers used for qRT-PCR and siRNA oligonucleotides. Click here for file
