# Supplementary figures and images for: Decreased expression of the long non-coding RNA FENDRR is associated with poor prognosis in gastric cancer and FENDRR regulates gastric cancer cell metastasis by affecting fibronectin1 expression
Source: J Hematol Oncol. 2014 Aug 29;7:63. doi: 10.1186/s13045-014-0063-7 (PMC4237812; doi:10.1186/s13045-014-0063-7)

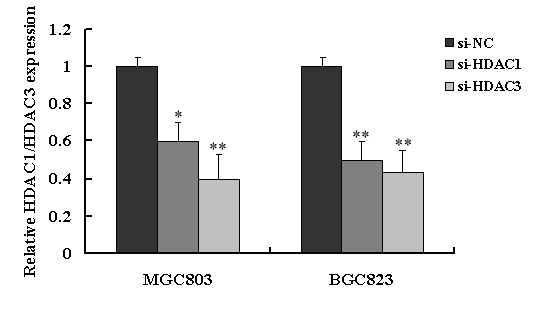

Supplement: Additional file 1: Figure S1. — qPCR analysis of HDAC1 and HDAC3 expression levels following the treatment of BGC823 and MGC803 cells with si-HDAC1 and si-HDAC3. Bars: SD; *p < 0.05, **p < 0.01. [file s13045-014-0063-7-S1.tiff]

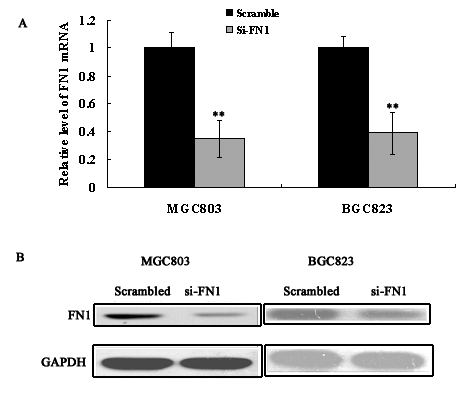

Supplement: Additional file 3: Figure S2. — MGC803 and BGC823 cells were transfected with an siRNA targeting FN1, causing the suppression of the mRNA (A) and protein levels (B) of FN1. Bars: SD; *p < 0.05, **p < 0.01. [file s13045-014-0063-7-S3.tiff]
